# Towards a versatile and economic Chagas Disease point-of-care testing system, by integrating loop-mediated isothermal amplification and contactless/label-free conductivity detection

**DOI:** 10.1371/journal.pntd.0009406

**Published:** 2021-05-14

**Authors:** Federico Figueredo, Fabiana Stolowicz, Adrián Vojnov, Wendell K. T. Coltro, Luciana Larocca, Carolina Carrillo, Eduardo Cortón

**Affiliations:** 1 Biological Chemistry Department, Science School and IQUIBICEN (FCEN–UBA-CONICET), Argentine; 2 Science and Technology Institute Cesar Milstein (ICT–Milstein–CONICET), Argentine; 3 Chemistry Institute, Federal University of Goiás, Campus Samambaia, Goiânia, Brazil; 4 National Institute of Science and Technology in Bioanalytics, Campinas, Brazil; University of Florida, UNITED STATES

## Abstract

Rapid diagnosis by using small, simple, and portable devices could represent one of the best strategies to limit the damage and contain the spread of viral, bacterial or protozoa diseases, principally when they can be transmitted by air and are highly contagious, as some respiratory viruses are. The presence of antibodies in blood or serum samples is not the best option for deciding when a person must be quarantined to stop transmission of disease, given that cured patients have antibodies, so the best diagnosis methods rely on the use of nucleic acid amplification procedures. Here we present a very simple device and detection principle, based on paper discs coupled to contactless conductivity (C^4^D) sensors, can provide fast and easy diagnostics that are needed when an epidemic outbreak develops. The paper device presented here solves one of the main drawbacks that nucleic acid amplification tests have when they are performed outside of central laboratories. As the device is sealed before amplification and integrally disposed in this way, amplimers release cannot occur, allowing repetitive testing in the physician’s practice, ambulances, or other places that are not prepared to avoid cross-contamination of new samples. The use of very low volume samples allows efficient reagent use and the development of low cost, simple, and disposable point**-**of**-**care diagnostic systems.

## Author summary

In 2005, the World Health Organization (WHO) recognized Chagas Disease as a neglected tropical disease. Meanwhile the serological tests, recommended by WHO, can be performed for chronic disease diagnosis, the nucleic acid amplification tests must be performed for the detection of the acute phase of the disease. Although the existing laboratory diagnosis tests for Chagas Disease are sensitive and highly reproducible, they cannot be performed in rural, low infrastructure environments, where this disease prevails. In this sense, the use of simple and portable analytical devices may be able to offer an affordable solution to this problem, allowing fast sampling, diagnosis and treatment prescription in one simple and fast intervention, as the performed by short term medical missions. In this study we show for the first time a diagnosis test comprising low cost materials and employing a contactless and label-free conductivity detection system that is used to read the result of a nucleic acid amplification reaction. The test showed high sensitivity for Chagas Disease diagnosis showing the potential to be used in rural and low income places.

## Introduction

In recent years, the diagnosis of infectious diseases has broken into a new and interesting phase. The goal is to implement simple, low-cost, and affordable technologies that complement a traditional central laboratory which has large, expensive, bench-top analyzers that need highly trained personnel. Commonly named point-of-care (POC) devices, these technologies are mainly developed to be used in emergent countries, where difficult living conditions and limited health care resources permit diseases to propagate more swiftly [1].

The following rigorous requirements are set for POC diagnosis devices: (a) rapid test results are needed in order to allow the patient to receive follow-up treatment; (b) to avoid false diagnostics, quantitative results should be accurate and comparable to the results from bench-top analyzers at central laboratories; and (c) they should be easy-to-use systems that could be run by non-experts with minimum user intervention [2]. Sensitive and accurate pathogen detection can be done by means of nucleic acid amplification analysis. As a way to detect a pathogen, DNA is frequently amplified by either the polymerase chain reaction (PCR) or by the loop mediated isothermal amplification reaction (LAMP). The latter is theoretically a better method for POC diagnosis for several reasons. LAMP can amplify a limited amount of DNA copies into millions of copies within an hour, it does not require a thermal cycling device since it operates isothermally at 60–65°C, it does not require highly trained personnel to conduct the reaction, and it shows remarkable performance in terms of sensitivity and stability [3].

As a result, LAMP has been efficiently implemented in paper-based platforms, because miniaturization typically enables shorter analysis times, reduces reagent consumption, minimizes risk of sample contamination, and often enhances assay performance [4,5]. A wide range of paper-based microfluidic designs have been developed. This is mostly related to the practicality of using these as POC diagnosis devices. Several studies have reported isothermal amplification in paper-based devices with endpoint optical detection by means of Au nanoparticles [6,7], fluorescent labels [8–11], or the color change of a colorimetric indicator [12,13]. In particular, visual interrogation performed using the naked eye leads to inaccurate quantification with low sensitivity [1,14].

Lateral flow tests are already used as POC devices to detect antigens or antibodies, and they have been recently adapted to detect the nucleic acid amplification products. Lateral flow tests based on Au nanoparticle aggregation are easy to read with the naked eye, but the main problem is that the reaction tube must be opened to place the strips inside, increasing the risk of cross-contamination with amplimers and obtaining false positives [15]. Paper-based diagnosis devices using molecular fluorescent labels usually increase the cost, add disposal problems (most of them are toxic), and present instability problems (photo bleaching). These are some common drawbacks previously reported elsewhere [16,17].

Electrochemical methods solve some of these aspects, and they can be applied in a miniaturized format more readily than optical methods [18]. They are based on the detection of electrochemically active species that bind to dsDNA and are measured through voltammetry-based techniques [19]. As the reaction progresses, the redox indicator binds to the dsDNA; the concentration of the free molecule in solution decreases and thus the redox current. Alternatively, other electrochemical methods add molybdate ions in solution to detect the phosphate ions generated during the LAMP reaction [20].

While electrochemical detection seems to be interesting for POC applications, electrode fouling with the components of the LAMP mixture and their biological components could be a problem that demands the use of disposable electrochemical cells; thus increasing the cost of the diagnosis test [21]. During the LAMP reaction, the conductivity of the solution changes, and this can be an indirect indicator of a positive result. Impedance detection can be used to monitor the amplification reaction with high sensitivity [22] using microelectrodes and microfluidic devices. The conductivity measurements show several advantages over optical and traditional electrochemical approaches for POC diagnosis since they are label**-**free. However, the electrodes are susceptible to the fouling effect, so they need to be disposed after one determination.

The capacitively coupled contactless conductivity detector (C^4^D) is based on the detection of the conductivity of a medium without physical contact between the electrodes and the measuring solution [23]. Since the electrodes are separated from the solution by an insulating material, unwanted side reactions such as electrolysis and corrosion, or fouling effects, are precluded [24,25]. Moreover, portable C^4^D analytical instruments have undergone significant development recently [26], showing the advantage to be suited for miniaturized analytical systems compared to optical-based detection methods. A few years ago, Gooding et al. [27] employed a C^4^D detector to monitor the LAMP reaction over time and measure changes in the sample’s conductivity. However, they used a pair of ring electrodes, placed outside glass tube walls, in an axial configuration which limit their application in planar analytical systems, as paper-based devices are [28]. Recently, planar C^4^D electrodes coupled to paper-based analytical devices have been studied for the detection of carbon dioxide gas [29] and soil conductivity [23], showing the potential for in field, portable and low cost applications.

In this study, we designed a POC paper-based diagnosis device based on isothermal amplification (LAMP) of *Trypanosoma cruzi* DNA using a C^4^D system to measure changes in the conductivity signal and thus obtain the endpoint result. *T*. *cruzi* is a protozoan parasite that causes Chagas Disease, a neglected tropical infection with a public health impact. It affects 8 million people worldwide; so, it is one of the biggest public health problems [30].

## Material and methods

### Reagents and biocomponents

Hydroxynaphthol blue (HNB) was obtained from Fluka; tris-HCl, KCl, MgSO_4_, (NH_4_)_2_SO_4_, Betaine, Triton X-100, Tween 20 and salmon sperm DNA (control DNA template) were purchased from Sigma-Aldrich. Bst DNA Polymerase Large Fragment was from New England Biolabs (USA), dNTPs was from INBIO Highway SA (Argentina), and the set of primers was from Macrogen (Korea). The LAMP reaction mix was prepared with tris-HCl (pH 8.8, 20 mmol L^-1^), KCl (10 mmol L^-1^), (NH_4_)_2_SO_4_ (10 mmol L^-1^), MgSO_4_ (8 mmol L^-1^), Betaine (800 mmol L^-1^), Tween 20 (0.1% p v^-1^), dNTP’s (1.4 mmol L^-1^), Bst DNA Polymerase (320 U mL^-1^), primers F3 and B3 (0.2 μmol L^-1^), LoopF and LoopB (0.8 μmol L^-1^), and FIP and BIP (1.6 μmol L^-1^).

### POC paper-based device fabrication

**Fig 1:**
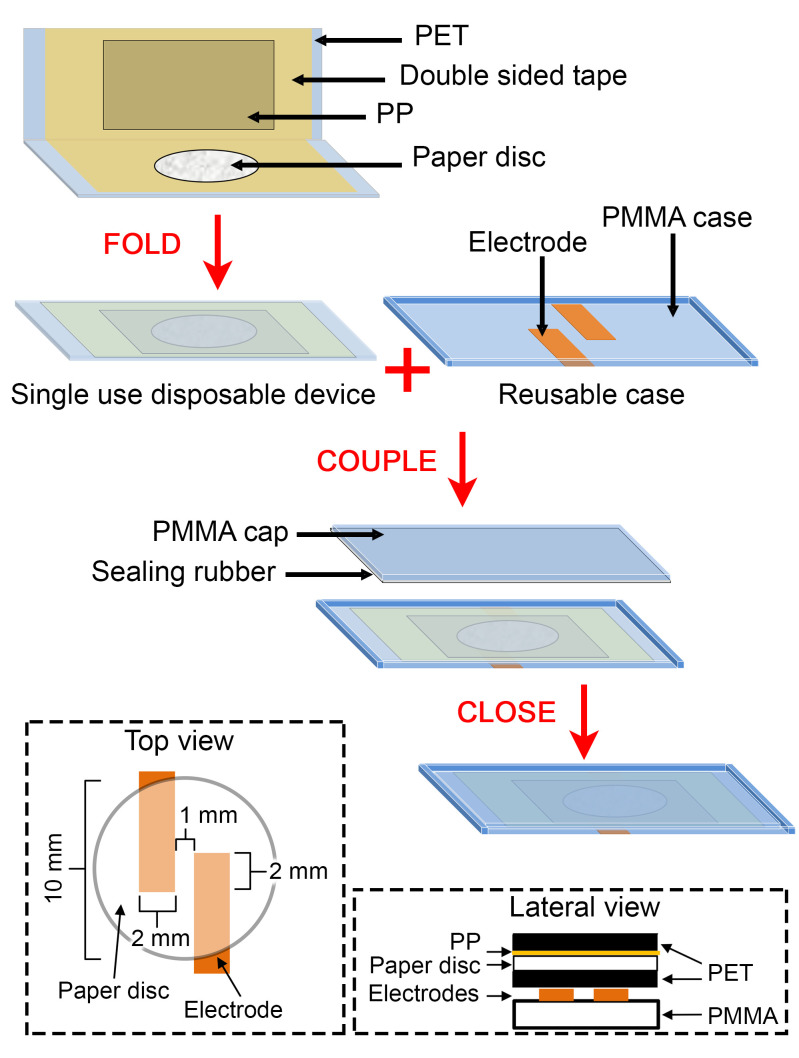
Design and fabrication of POC analytical device. Schematic representation showing the materials comprising the single use disposable device and the reusable case. Inset pictures display the top and the lateral view of the assembled POC analytical device showing the electrode and paper disc disposition in detail.

The analytical assay was performed with a single use disposable device and a reusable case, containing the electrodes. The disposable device was fabricated by placing a disc of Whatman grade 1 paper with a diameter of 10 mm, and a polypropylene film (PP), over a 150 μm poly(ethylene terephthalate) (PET) layer, with the aid of a double sided adhesive tape (Fig 1). For the reusable case fabrication, a 4 mm thickness poly(methyl methacrylate) (PMMA) sheet was engraved with a CNC machine (Modela MDX-20 Roland, Shizuoka-ken, Japan) to produce the 30 x 15-mm PMMA case designed as a pocket that hold the disposable device. Two sensing electrodes made with copper adhesive tape were at the base of the PMMA case. The electrodes (2 mm wide and 14 mm long) were arranged in an antiparallel configuration keeping a gap of 1 mm between them (Fig 1). After the disposable device was inserted inside the PMMA case pocket, the resulting distance between the electrodes layer and the paper disc layer was 150 μm. A PMMA cap with a sealing rubber film was fabricated to close the analytical device that was sealed the aid of two clamps.

### LAMP reaction in paper disc and HNB detection

HNB, a colorimetric reagent previously used to follow LAMP reactions [31,32], was used at a final concentration of 120 μmol L^-1^ in the reaction mixture containing the DNA template or without it (called non-template control or NTC). The reaction mix with HNB was loaded on the paper disc inside the analytical device and, once closed, it was heated to 65°C for 5, 15, 30, and 60 min of incubation. At each time, the paper disc was irradiated at 540 nm, and the fluorescence emission signal was acquired and analyzed with an image acquisition system and software (Amersham Imager 600, GE Healthcare, Tokyo, Japan). The signal intensity of the paper disc was measured at zero**-**point signal (I_0_) and at each detection time (I), the signal intensity ratio was calculated following a previously reported procedure (10).

### Contactless conductivity measurement (C^4^D)

DNA amplification reactions (in paper discs) were detected using a homemade C^4^D module comprising both the current-to-voltage signal converter and signal treatment circuits. It was constructed according to Silva et al. [33]. The entire homemade C^4^D detection system uses a sinusoidal wave provided by a function generator (model DS335, SRS Stanford Research Instruments, California, USA) that goes through the excitation electrode. The receiver electrode is connected to the C^4^D detection module, and then to an ADC converter (National Instruments, NI USB-6009) allowing digitalization and computer data acquisition by means of a LabVIEW-based software with a time resolution of 1 ms. Optimization experiments were performed by applying a 400, 500, or 600 kHz sinusoidal wave with 1 V_pp_ amplitude to the excitation electrode.

### LAMP reaction in paper disc and C^4^D detection

**Fig 2:**
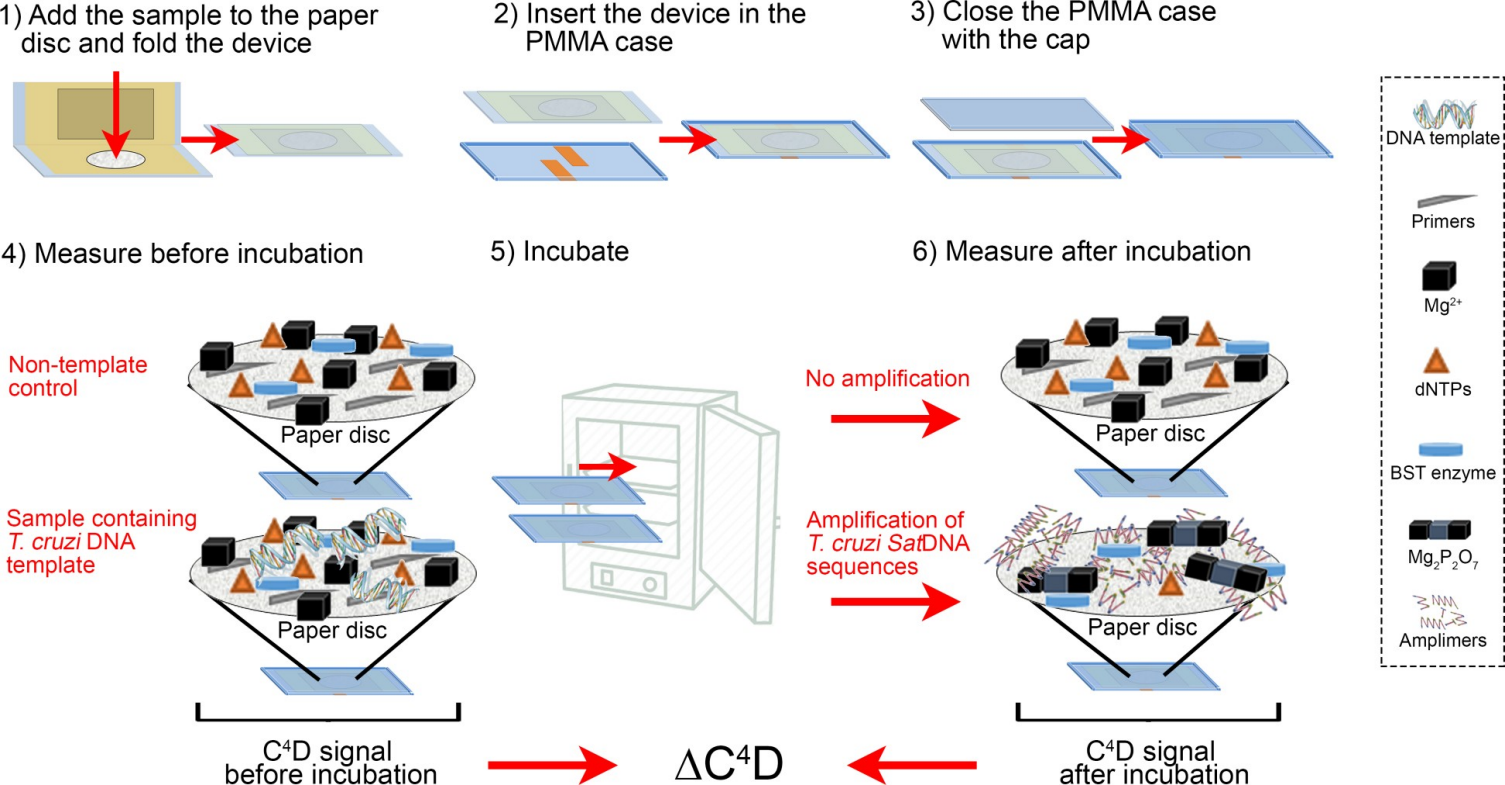
LAMP reaction in paper disc and C^4^D measurement. Schematic illustration showing the steps required to perform the LAMP amplification and the conductivity detection for NTC and samples containing *T*. *cruzi* DNA template.

In this study, we used LAMP solution reagents containing primers whose design was based on the repetitive satellite DNA sequences (SatDNA) of *T*. *cruzi*. SatDNA is the most abundant repetitive sequence in the *T*. *cruzi* genome, composed by 10^5^ copies of a 195**-**nucleotide repeat [34]. The primers were designed *in silico* and tested performing LAMP reactions using 1 pg of genomic DNA from *T*. *cruzi* CL Brener. As we expected due to the use of conserved sequences for the design of the primers, the reaction amplified with the template of all of the Discrete Typing Units (DTU) from I to VI (provided by Dr. Schijman, INGEBI–CONICET, Buenos Aires, Argentina), and the typical pattern of stepped bands were visualized with agarose gel electrophoresis technique. All the experiments performed in this study were done with CL Brener DTU VI. The nucleotide sequence of the primers used in the study (F3, B3, LoopB, LoopF, FIP and BIP) are not provided, since the trial is being developed into a commercial product. In a typical assay, 11 μL of the LAMP reaction mix was loaded at the center of a paper disc (Fig 1, single use disposable device), as the negative control (non-template control, NTC). On the other hand, the device was loaded with 11 μL of the LAMP reaction mix but containing 3.2, 0.32, or 0.032 fg of the DNA template (genomic DNA of *T*. *cruzi*). Then, the single use disposable device was immediately folded and placed inside the PMMA case pocket and closed with the PMMA cap (Fig 2). The closed PMMA case containing the disposable device inside was connected to C^4^D electronics, and the signal was acquired in real time until stabilization (signal slope < 1 mV min^-1^). Then, the entire device was placed at 65°C for 60 min. After that, it was connected again to C^4^D electronics, and the signal was acquired following the same criteria used before. The result of the assay arises from a comparison between the signals obtained before and after incubation as follows:

$$\Delta C^{4}D=C^{4}D\;signal\;\left(after\;incubation\right)‐C^{4}D\;signal\;\left(before\;incubation\right)$$ (1)

Where “C^4^D signal (after incubation)” is the signal value obtained with the C^4^D after incubation and “C^4^D signal (before incubation)” is the signal value obtained with C^4^D before incubation.

### LAMP reaction in solution

The LAMP reaction was simultaneously performed in solution using the same reagents and conditions employed in this study. Microtubes containing 60 μL of the sample mix, NTC, or DNA template were incubated for 60 min at 65°C (n = 3). After the reaction, the loading buffer (0.05% w v^-1^ bromophenol blue, 5% w v^-1^ glycerol) and 10 μL of the LAMP amplification product were mixed, placed in a standard TAE-1.5% agarose gel electrophoresis containing Sybr Safe, and run for 50 min at 50 V (Mupid EXU). The gel was revealed under a UV analyzer (Gel Doc EZ Imager, Bio-Rad). In another set of experiments, LAMP assays were performed using the same conditions described here but adding HNB (120 μmol L^-1^) to the sample mix. The results were observed by the naked eye and recorded with a digital camera.

### LAMP reaction in paper disc analyzed with gel electrophoresis

Gel electrophoresis analysis techniques were employed to detect the LAMP amplification products obtained after a typical reaction the POC analytical device. DNA was eluted from paper discs employing the protocol described here, which was based on previously reported protocols [35,36]. Immediately after the amplification reaction, the paper disc was immersed in 60 μL of ultrapure water (resistivity greater than 18.2 MΩ) and incubated for 2 min at 100°C; then the paper disc was squeezed with a pipette tip and the entire volume was analyzed with gel electrophoresis using the methodology described before (see section: “LAMP reaction in solution” in Material and methods).

## Results

### LAMP reaction in paper discs and HNB detection

**Fig 3:**
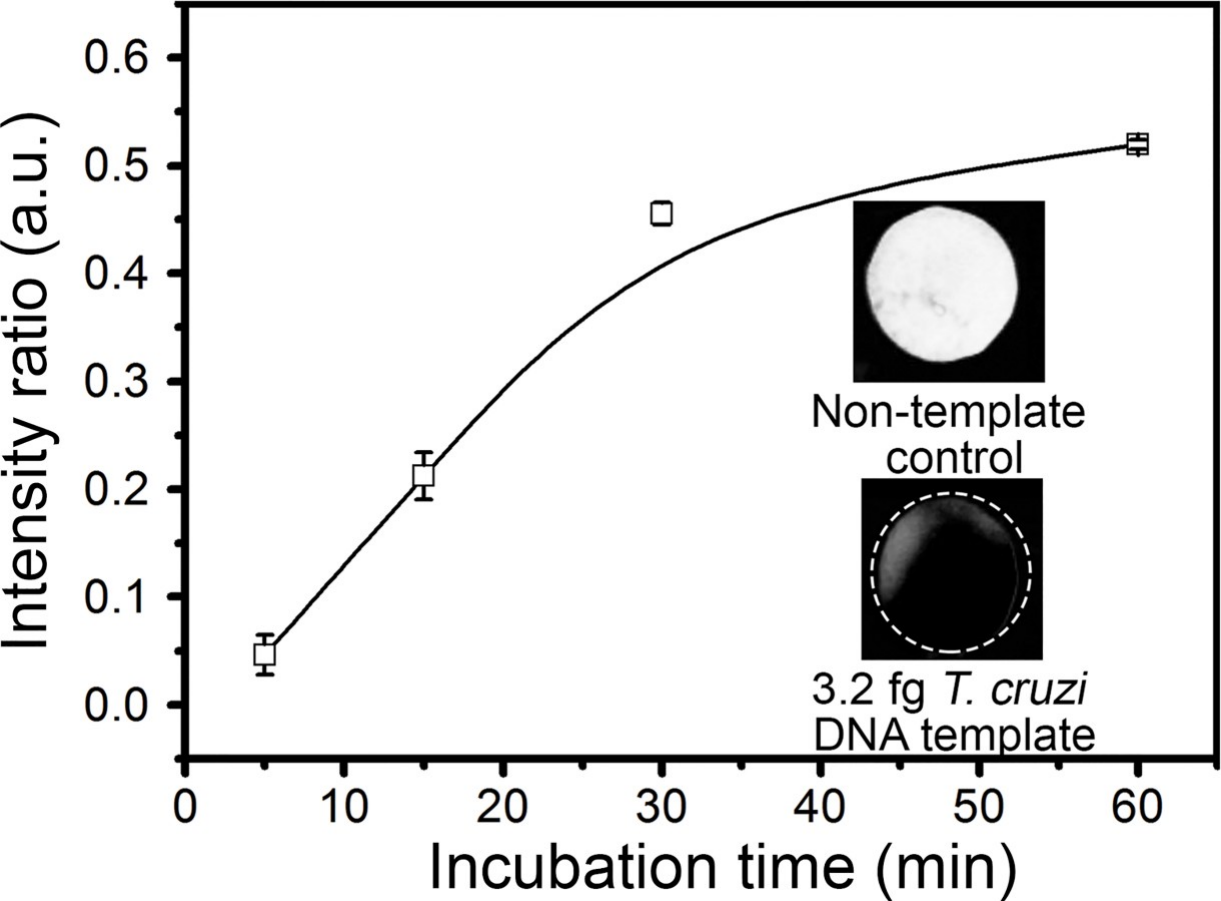
LAMP reaction in paper discs followed by HNB fluorescence. The LAMP reaction containing 3.2 fg of *T*. *cruzi* DNA template was incubated at 65°C. Paper discs were extracted from the device at different incubation times (0, 5, 15, 30, and 60 min), irradiated at 540 nm, and pictures were taken using a 620 nm emission filter. The plot shows the analytical curve obtained from the signal intensity ratio as function of reaction time (n = 3). Pictures included in the plot show the results obtained for non-template control (NTC) and samples containing 3.2 fg of template, after 60 min of incubation.

HNB has been used as a colorimetric dye for isothermal amplification [32], and it is analyzed with the naked eye. Kim et al. [10] reported that the fluorescence patron of HNB changes in a Mg^2+^-dependent way: when LAMP reaction makes progress, pyrophosphate ion is generated as a by-product and reacts with Mg^2+^; then the HNB fluorescence signal decreases. In the LAMP reaction performed here using 3.2 fg of DNA template, the highest fluorescence signal for HNB was obtained at the initial reaction time (0 min), and it decreased progressively to the minimal signal at the end of the assay (Fig 3). With the naked eye, differences in HNB color were not observed on the paper disc. Fluorescence changes were easily noted for up to 30 min; however, the quantified signal intensity curve reached the highest value at 60 min, indicating the reaction progressed until this time, similar to the results obtained by Kim et al. [10].

### Contactless conductivity measurement (C^4^D) in paper discs

**Fig 4:**
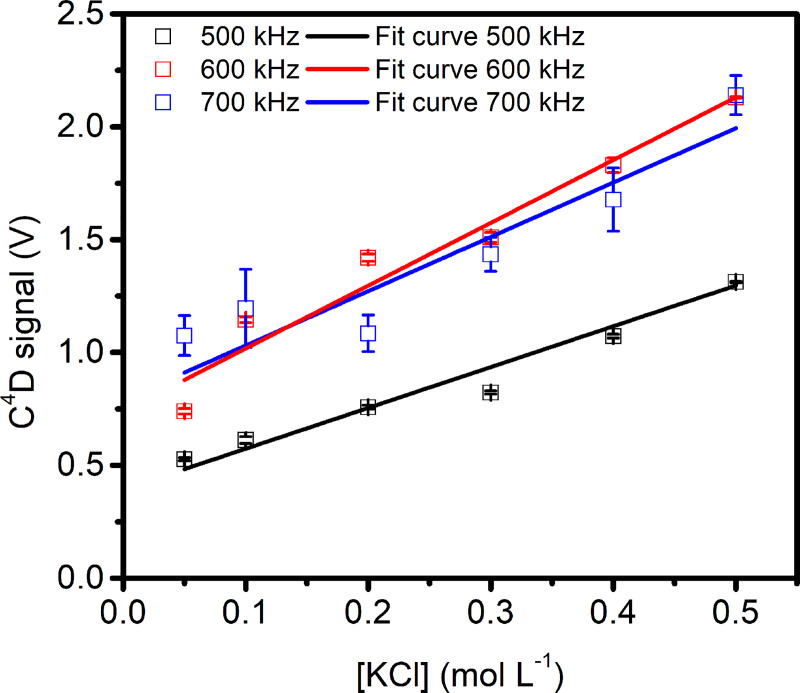
C^4^D signals as a function of the solution concentration (related to conductivity). The conductivity was recorded at room temperature and frequencies of 500, 600, and 700 kHz. The lines represent the lineal regression of each data set (n = 3).

To find the best instrumental conditions (basically good sensitivity and low noise), we performed conductivity measurements using our paper-based set-up, and KCl solutions (0.05, 0.1, 0.2, 0.3, 0.4, and 0.5 mol L^-1^) were normally used as conductivity standards. The POC analytical device was characterized using frequencies of 500, 600, and 700 kHz and 1 V_pp_. Experiments were done at 20–25°C using one paper disc for each KCl concentration. For the three frequencies used, there was a positive relationship between solution ionic strength and C^4^D (V) output (Fig 4). The linear regression fit the experimental data well with R^2^ values of 0.973, 0.981, and 0.836 for frequencies of 500, 600, and 700 kHz, respectively. However, the 600 kHz frequency showed better characteristics, higher sensitivity (higher slope) and lower deviations (small error bars). So, this frequency was selected for further experiments.

### LAMP reaction in paper disc and C^4^D detection

**Fig 5:**
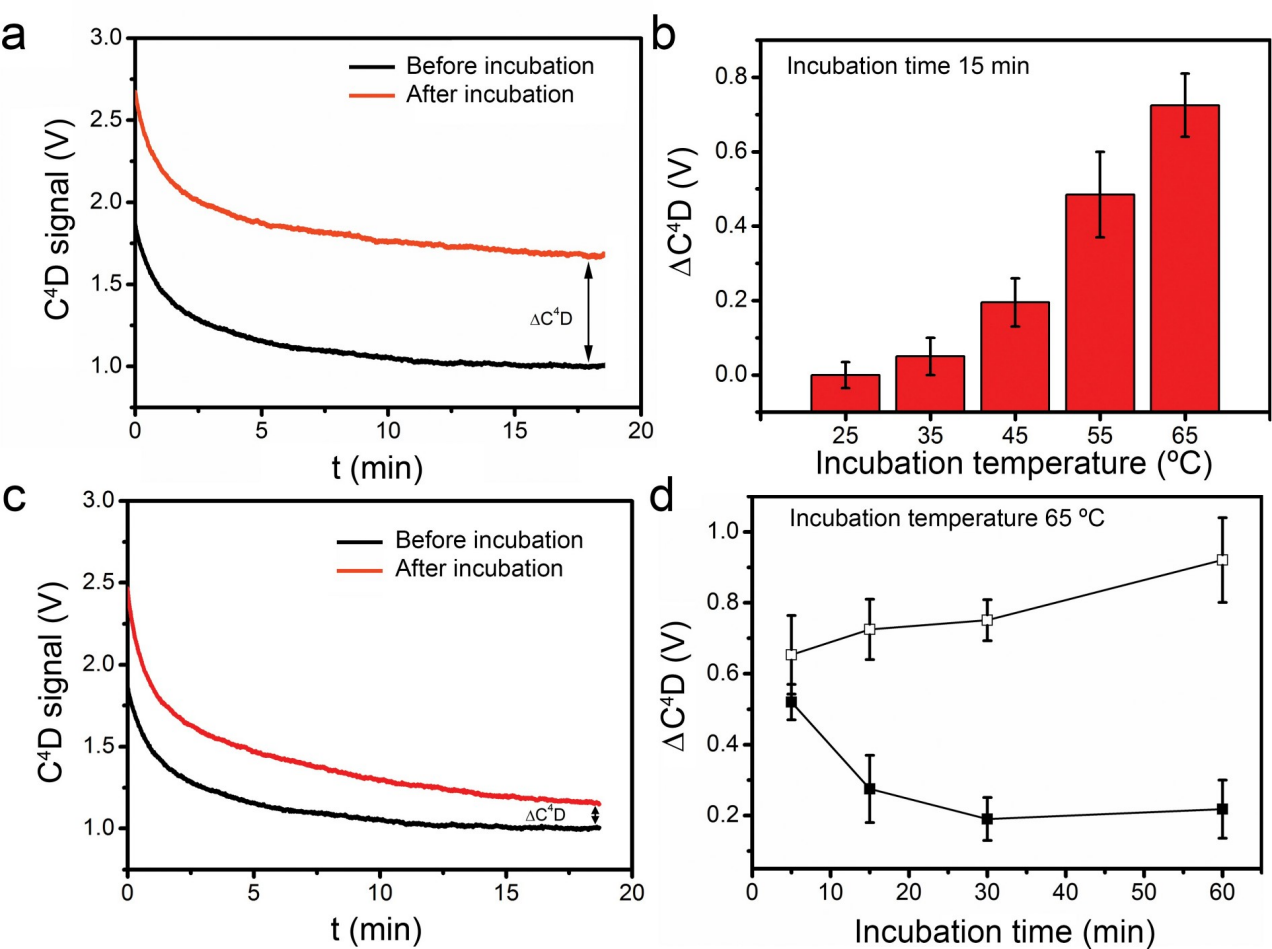
C^4^D signal recorded from paper discs under different LAMP conditions. The real time C^4^D signal was measured at room temperature before incubation and after incubation at the indicated temperatures and times. (a) C^4^D signal displayed by NTC samples, before and after incubation at 65°C for 60 min. (b) Differential conductivity signal (ΔC^4^D) of NTC samples before and after incubation at different temperatures for 15 min. (c) C^4^D signal displayed by samples containing 3.2 fg of *T*. *cruzi* DNA template before and after incubation at 65°C for 60 min. (d) ΔC^4^D curves of (□) NTC and (■) samples containing 3.2 fg of *T*. *cruzi* DNA template measured before and after different times of incubation at 65°C.

Once the LAMP reaction was performed, the first C^4^D determinations were done using a non-template control (NTC) containing all of the LAMP reagents except the *T*. *cruzi* DNA template. The C^4^D signal was recorded in real time for 20 min at room temperature (20–25°C) before and then after the incubation step (60 min at 65°C). In these conditions, the C^4^D signal recorded after the incubation was higher than that observed before incubation (Fig 5A). As an additional internal control, similar experiments were done by replacing the LAMP mix with a KCl solution, and no changes in the C^4^D signal were detected between samples before and after the incubation (RSD < 5%).

Aiming to investigate the effect of incubation temperature on the acquired C^4^D signal, we performed short experiments (15 min) using NTC samples at 25, 35, 45, 55 and 65°C. In all cases, the C^4^D signal was recorded at room temperature (20–25°C) and the differential C^4^D signal (ΔC^4^D signal) was determined (see section: “LAMP reaction in paper disc and C^4^D detection” in Material and methods). A direct relationship was observed between the incubation temperature and the ΔC^4^D signal; this behavior is independent from any amplification process, since the assay was performed in NTC (Fig 5B). In the assay, higher incubation temperatures seemed to favor better desorption of the biomolecules from the paper fiber to the mobile phase, showing higher C^4^D conductivity values. Having tested the effect of incubation temperature on the C^4^D signal, the temperature was set at 65°C for next experiments. Thereafter, the C^4^D signal was recorded in a complete LAMP reaction. The amplification was performed at 65°C for 60 min with 3.2 fg of DNA template, and the C^4^D signal before and after the incubation was taken by real time curves at room temperature (Fig 5C). The C^4^D signal of the positive LAMP sample after the amplification was higher than before but notably lower than the signal obtained after incubation in NTC conditions (Fig 5A vs. 5C). In fact, samples containing DNA template showed conductivity values similar to NTC controls during the first 5 min of incubation, but later, as amplification proceeded, the conductivity signal showed clear differences between both conditions (Fig 5D). In this sense, the ΔC^4^D signal was progressively decreasing in the positive LAMP reaction. This effect can be attributed to the consumption of high conductivity reagents (dNTPs and Mg^2+^) and the generation of low conductivity products such as (DNA)_n_ and magnesium pyrophosphate [21,27], as shown in these equations:

$${\left(DNA\right)}_{n‐1}+dNTP\to {\left(DNA\right)}_{n}+P_{2}O_{7}{}^{4‐}+2H^{+}$$ (2)

$$P_{2}O_{7}{}^{4‐}+2Mg^{2+}\to Mg_{2}P_{2}O_{7}$$ (3)

$$2Tris+2H^{+}\leftarrow \to 2Tris‐H^{+}$$ (4)

Considering the ΔC^4^D values obtained in NTC and positive samples, we defined 60 min as the LAMP incubation time required for an accurate read-out, since value differences were clearer than those at shorter incubation times (Fig 5D).

**Fig 6:**
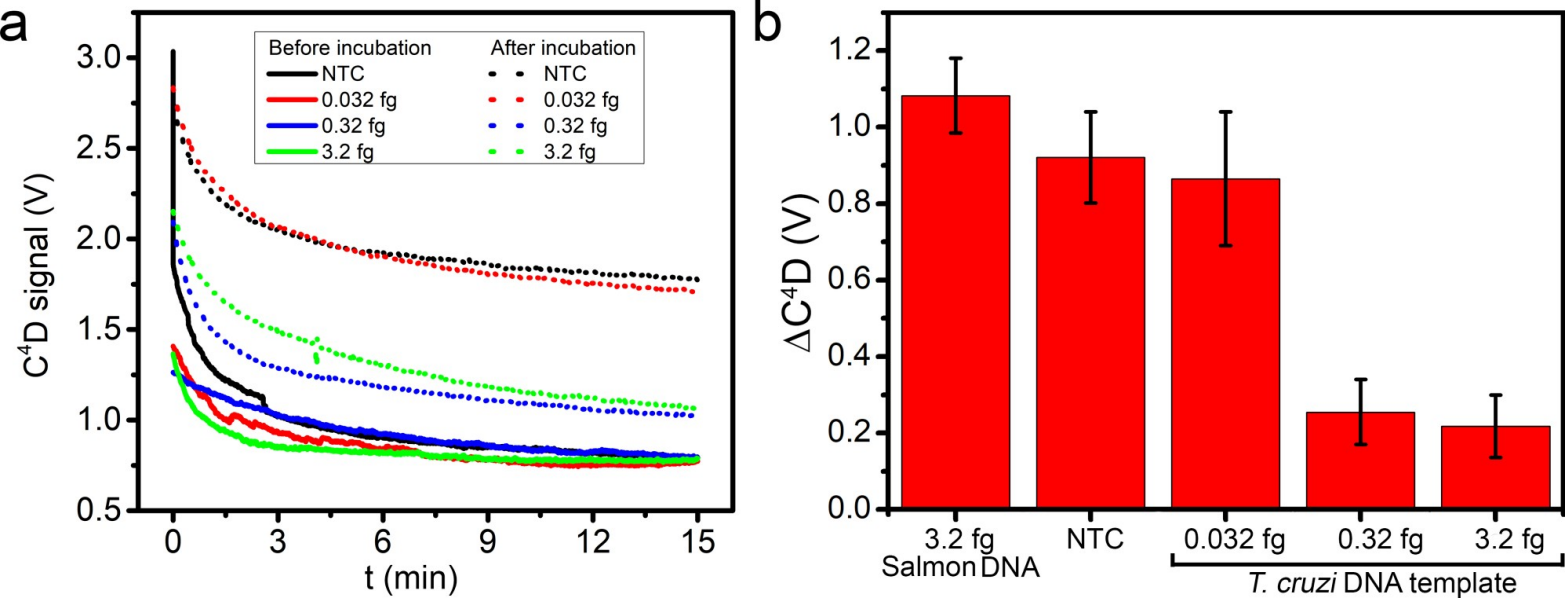
Detection sensitivity of LAMP amplification by the designed device. (a) Representative C^4^D raw signals obtained before and after incubation with NTC and samples containing 0.032, 0.32 and 3.2 fg of *T*. *cruzi* DNA template. (b) ΔC^4^D signal obtained for 3.2 fg of a heterologous template (salmon DNA), NTC, and for 0.032, 0.32 and 3.2 fg of *T*. *cruzi* DNA template (n = 3).

Then we assayed the detection sensitivity by measuring the ΔC^4^D signal before and after incubation at 65°C for 60 min using serial dilutions of *T*. *cruzi* DNA as template (Fig 6A). Besides the NTC reaction, a LAMP reaction with heterologous DNA (salmon sperm DNA, “Salmon DNA”) was prepared as an additional control to check the possible effect in the ΔC^4^D signal (Fig 6B). The developed analytical device could clearly detect 3.2 fg and 0.32 fg of the specific *T*. *cruzi* DNA template (n = 3), which represents 1–5 copies of the target gene per reaction [37,38]. Samples containing 0.032 fg of *T*. *cruzi* DNA template were not detected, they showed ΔC^4^D values similar to those obtained for NTC and heterologous DNA (Salmon DNA) samples, where amplification did not succeed.

### LAMP reaction in solution

LAMP reactions in solution were done maintaining the same conditions and using the same reagent employed for the analytical device designed and studied in this article. Amplification reactions were conducted with solutions containing all of the sample mix reagents and adding 1 pg of DNA template. The results of the electrophoresis gel are presented in Electronic Supplementary Material (ESM) S1A Fig. When the electrophoresis gel was analyzed, we observed the typical stepped band pattern of the LAMP reaction (n = 3) for the positive reaction (1 pg of genomic DNA template), while no bands were seen in negative controls. Further analysis was done when HNB was added to the reaction solution; the typical blue color was obtained for positive samples and the violet color was obtained for NTC samples (ESM S1B Fig). These results allowed us to confirm that the LAMP reaction components (enzymes, dNTP´s and primers) are working, and the amplification reaction occurs.

### LAMP reaction in paper disc analyzed with gel electrophoresis

LAMP reaction was conducted in paper discs similarly to experiments performed in this article. At the end of the assay, DNA was eluted and agarose gel electrophoresis was used to reveal the amplification product. However, the typical stepped band pattern was not observed in gel electrophoresis when positive samples (containing 3.5 fg of *T*. *cruzi* DNA template) were analyzed. While gel electrophoresis is a gold standard technique to reveal amplification products, the limit of detection of the technique is probably not enough to detect the amount of amplimers produced in the paper disc. Then, LAMP reactions in solution containing different amounts of template were conducted to evaluate the gel electrophoresis sensitivity as a reference technique. The results showed that when 10 fg or even lower amounts of DNA template were used to perform the reaction in solution, it was not possible to appreciate the typical LAMP pattern (as shown in ESM S1A Fig). Therefore, if we extrapolate the results obtained in 60 μL solution with 10 fg of *T*. *cruzi* DNA template to those results obtained for paper-disc containing 11 μL solution with 3.5 fg of *T*. *cruzi* DNA, we conclude that the absence of a band is mainly because the limit of detection was not good enough for detecting the amplimers generated with the reagents and conditions employed in this article.

## Discussion

The amplification reactions in paper-based analytical devices were studied employing HNB and fluorescence analytical methods to detect the reaction product. As seen in the results obtained, the entire analytical device can be successfully used for the amplification of *T*. *cruzi* DNA template. When the analytical device was coupled with the C^4^D detector, the conductivity signals were studied. First, we used KCl solutions to evaluate which frequency provide higher sensitivity and lower deviation. Once this parameter was set (600 kHz), amplification assays were performed. Preliminary experiment showed that the incubation temperature affect in some way the conductivity signal obtained at room temperature. The results showed that independently from any amplification reaction, as is seen for NTC, after the incubation step, the conductivity signal increase. This behavior can be explained by an unspecific adsorption of sample biomolecules such as primers, dNTPs, and enzymes on paper fibers at room temperature. Cellulose fibers contain hydroxyl groups which are capable of forming strong hydrogen bonds which result in powerful intermolecular attractions [35,39] The intermolecular forces between cellulose fibers and biomolecules diminish when they are subject to heat treatment, which acts as a physical elution agent. This has been shown particularly for nucleic acid samples in FTA-cards or cellulose discs [36,40]. Moreover, the heat-desorbed biomolecules are still retained in the mobile phase even when the paper disc drops to room temperature, as shown by C^4^D conductivity measurements for NTC samples in Fig 5B. These explain why the conductivity signal are always higher after the incubation step. Meanwhile the temperature effect was studied to understand the results obtained in a typical amplification assay, the LAMP reaction should be perform at temperatures of 65°C to achieve the best amplification efficiency. Consequently, the following experiments were done at 65°C.

The real-time PCR (qPCR) is one of the most versatile and widely used methods in molecular biology and clinical diagnosis, mainly because of its high sensitivity, and good accuracy. In particular, for *T*. *cruzi* detection, there is a lack of well stablished experimental procedures associated to qPCR detection. However, among the previously reported studies, the minimum quantity of *T*. *cruzi* DNA detected with qPCR was between 0.13 and 3 fg per reaction [41,42], with variation associated mainly to the experimental procedure employed, as was recently reviewed and discussed [43]. In this study, we presented a POC analytical assay that can be employed to detect 0.32 fg of *T*. *cruzi* DNA, which is as low as those DNA amounts detected with the qPCR, but with several advantages associated mainly to POC assays. The contactless conductivity assay presented here is reagent-less (additional reagent for the detection are not needed), label**-**free (primers do not need to be labelled), and the incubation and detection is performed in the same disposable device avoiding any possibility of cross contamination. The amount of DNA template that the contactless conductivity assay of this study detected is similar to that reported by Zhang et al. [27], who could detect 0.51 fg of DNA template in 30 min with a C^4^D system, in a greater volume of sample (200 μL), and glass tubes with very thin walls commonly used for nuclear magnetic resonance studies. Further, the proposed assay can detect DNA concentrations lower than other POC devices based on LAMP used for the detection of other analytical targets (such as bacteria). These comparisons applies to those paper-based devices using HNB fluorescence [10] or color [44] change detection, and lateral flow devices [7]. In fact, in previous works, HNB fluorescence change detection of the positive LAMP reaction in paper-based devices could detect 0.7 pg (4.1 *x* 10^2^ copies) of *Streptococcus pneumoniae* genomic DNA [10], while a paper-plastic hybrid device detected 18 fg of *Staphylococcus aureus* DNA with HNB color change detection by the naked eye [44].

## Conclusion

A novel POC device was developed using simple and inexpensive materials. The feasibility of the proposed device to diagnose Chagas Disease employing a nucleic acid amplification procedure was successfully demonstrated, thus revealing great potential for POC molecular diagnostics. As the detection principle is generic for most nucleic acid amplification tests (the change of solution conductivity arising from the enzymatic polymerization reaction), the POC device could probably be used to run other diagnosis reactions. To fully corroborate the device performance, Salmon DNA was used as a control, and a low concentration of target nucleic acid template was assayed, showing excellent sensitivity. The detection method proposed was compared to colorimetric detection, which is typically used to develop POC devices, showing good agreement. We demonstrated that sensitive conductivity measurements using disposable single use devices allowed us to develop a POC molecular assay useful for diagnosing *T*. *cruzi* parasitosis, given the detection limit obtained of 0.32 fg with the *T*. *cruzi* DNA template, which represents 1–5 copies of the target gene/s per reaction. As we use simple low cost materials, and minimal reagent volume, the proposed POC device can overcome other POC methods when cost, easy disposability and biosafety aspects are considered, as the device did not include sharp or glass materials, and is designed to be disposed closed. In field applications, where temperatures can reach 30–35°C, the baseline signal coming from the C^4^D can be potentially affected. However, as control samples must be run simultaneously with the suspected sample, background variations will be most probably cancelled. Meanwhile this is an approximation, we believe that any potential scenario related to field conditions need to be tested further. To really become an integrated POC system, the device should work with a drop of human blood, and designed as an entire portable detection system including the analytical device, the detection system and the incubator. Future investigations should be performed to study the possibility to use blood samples as such, and evaluate if any type of sample treatment need to be done.

## Supporting information

S1 Fig LAMP reaction revealed by alternatives methods. (a) Agarose gel electrophoresis and (b) HNB colorimetric reaction. Positives samples contain 1 pg of genomic DNA of *T*. *cruzi*.
(TIF)
