# Supplementary figures and images for: Towards a versatile and economic Chagas Disease point-of-care testing system, by integrating loop-mediated isothermal amplification and contactless/label-free conductivity detection
Source: PLoS Negl Trop Dis. 2021 May 14;15(5):e0009406. doi: 10.1371/journal.pntd.0009406 (PMC8153438; doi:10.1371/journal.pntd.0009406)

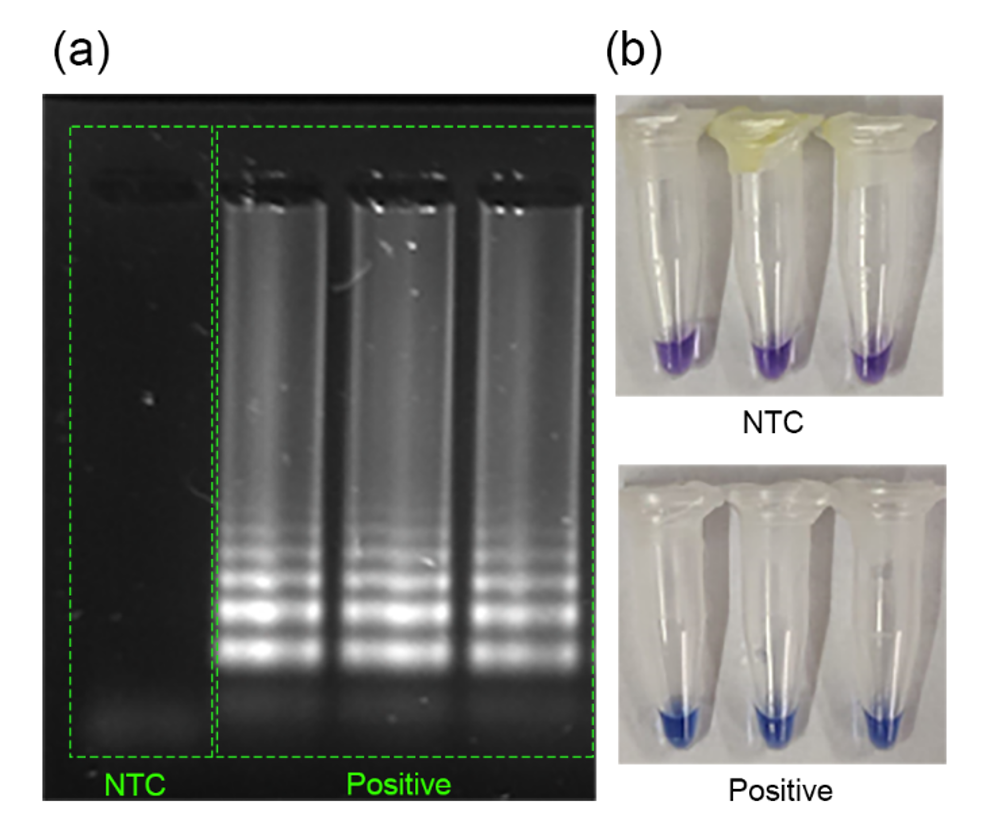

Supplement: S1 Fig — (a) Agarose gel electrophoresis and (b) HNB colorimetric reaction. Positives samples contain 1 pg of genomic DNA of T. cruzi. (TIF) [file pntd.0009406.s001.tif]
